# Prevalence and correlates of underweight among lactating mothers aged 20–49 years living in the Urban Slums of Kampala City, Uganda: A mixed-methods cross-sectional study

**DOI:** 10.1371/journal.pgph.0007389

**Published:** 2026-09-29

**Authors:** Saharu Magona Nerima, Jireh Kirabo, Edirisa Juniour Nsubuga, Grace Nanyombi, Joel J. Komakech, Latiffu Musisi, Joseph Mbabazi, John Bosco Ddamulira

**Affiliations:** 1 Department of Community Health and Behavioral Sciences, Makerere University School of Public Health, Kampala, Uganda; 2 Maternal Child Health Department, C-Care International Hospital Kampala (C-Care IHK), Kampala, Uganda; 3 Department of Biochemistry, Nutrition, and Health Promotion, Mississippi State University, Starkville, Mississippi, United States of America; 4 Department of Family and Consumer Sciences, North Carolina Agricultural & Technical State University, Greensboro, North Carolina, United States of America; 5 Department of Health Sciences, Uganda Martyrs University, Nkozi, Uganda; 6 Department of Epidemiology and Biostatistics (Epi-Bio), Makerere University School of Public Health, Kampala, Uganda; PLOS: Public Library of Science, UNITED STATES OF AMERICA

## Abstract

Suboptimal nutrient intake during lactation contributes to maternal underweight and reduced breastmilk quantity and quality, with potentially severe consequences for maternal and infant health. Although underweight prevalence among lactating mothers in urban slums is presumed higher compared to other areas, empirical data from Kampala’s slums remains limited. This study examined the prevalence and correlates of underweight among lactating mothers aged 20–49 years in Kampala’s slums. A convergent mixed methods cross-sectional study was conducted among 285 lactating mothers in Kampala’s slums. Multivariable modified Poisson regression generated adjusted prevalence ratios (aPRs), with statistical significance at *p* < 0.05. A prespecified multivariable modified Poisson regression model guided by the UNICEF conceptual framework and literature was fitted to identify correlates of underweight among lactating mothers. Model robustness was assessed through sensitivity analyses using three distinct covariate blocks. Ten key informant interviews with health workers explored perceptions of factors associated with higher and lower prevalence of maternal underweight. Inductive thematic content analysis was used for the qualitative data whose findings were triangulated with quantitative results for a comprehensive and better contextual understanding of findings. The average age of the lactating mothers was 29 years (standard deviation = 5). Underweight (13.7%) among lactating mothers was moderately prevalent. Correlates of underweight among lactating mothers were household food insecurity (aPR = 1.4[1.2-1.6]), household size of <5 members (aPR = 0.94[0.89-0.99]), meal frequency of ≥3 meals/day (aPR = 0.74[0.66-0.83]), and minimum dietary diversity for women (MDD‑W) (aPR = 0.91[0.84-0.96]). Results from sensitivity analyses supported robustness of the findings. Factors perceived to be associated with higher prevalence of maternal underweight included household food insecurity and working mothers. Factors perceived to be associated with lower prevalence of maternal underweight included higher meal frequency, meeting MDD‑W and fewer household members. Community sensitization on family planning, household food security, meal frequency, and dietary diversity through integrated community outreaches could improve nutritional status of lactating mothers.

## Introduction

The World Health Organization (WHO) defines malnutrition as imbalances in a person’s intake of energy and/or nutrients [1]. Malnutrition is divided into three broad categories of undernutrition (underweight, wasting, and stunting), overnutrition (overweight, obesity, and excesses and toxicities in vitamins and minerals) and micronutrient deficiencies (deficiency in vitamins and minerals) [1]. Underweight among lactating mothers is defined as too low body weight relative to height for an average healthy adult woman. According to the WHO, underweight is defined as a body mass index (BMI) of  < 18.5 kg/m^2^ for women aged 20–49 years [1,2]. Globally, an estimated 154 million (10%) of all women of child bearing age in the ages of 20–49 years were underweight, with 68% of whom were from South Asia and sub-Saharan Africa [3]. Within Eastern and Southern Africa 10 million women of child bearing age in the ages of 20–49 years were underweight [3]. In Uganda, underweight prevalence reached 9% among women of reproductive age [4] and approximately 8% among lactating mothers aged 20–49 years [5].

Undernutrition in all its forms is a direct result of inadequate food intake; and excess nutrient utilization and excretion due to disease, infection and other metabolic abnormalities [1]. Previous studies showed that educational status, nutritional knowledge, dietary practice, marital status [5,6], maternal age [7], family size [8], birth to pregnancy interval, and postnatal care [9] were associated with underweight in lactating mothers. Lactation is a high-risk period in a mother’s life resulting in increased physiological demands due to the extra nutrient requirements accounting for 8% of total household food energy requirements [10]. Unmet maternal nutrient demands during lactation have been reported to cause maternal underweight, which reduces breast milk quantity and quality, increasing the risk of child morbidity, mortality, and long-term adverse health outcomes [11,12]. Maternal underweight during lactation is associated with adverse health outcomes for both mothers and their infants health, including compromised maternal recovery, impaired infant and young child growth and development [1,12]. Additionally, maternal underweight is associated with childhood stunting [13], compromised maternal cognitive and physical function, which subsequently leads to inadequate childcare, and reduced quality of maternal-infant interaction [14]. Consequently, malnutrition and its effects are passed down from generation to generation, perpetuating the vicious cycle of malnutrition [15]. Maternal underweight during lactation can be prevented and managed through several ways including but not limited to nutritional counseling and social support; dietary diversification (daily intake of fruits, vegetables, and animal products); intake of fortified foods (e.g., iodized salt, and vitamin A fortified cooking oils); micronutrient supplementation (either folic acid, iron, and or multiple micronutrients); increased food intake through consumption of the equivalent of at least two extra meals per day by the breastfeeding mothers providing an extra ~550 kcal/day on top of the recommended dietary allowance (RDA); deworming; intake of biofortified foods (e.g., yellow/orange sweet potatoes, and iron rich beans); and getting enough rest [16–18].

Urban slums are densely populated areas designated by governments or local health authorities, typically based on census data or geographic boundaries [19]. These slums are characterized by inadequate housing, poor sanitation, and limited access to essential services such as clean water and healthcare [19–21]. Residents comprise highly vulnerable and marginalized populations who often lack basic necessities, experience poverty, unemployment, and suffer poor health outcomes resulting from poor living conditions and limited socioeconomic opportunities [19–21]. Kampala’s urban slums reflect global patterns with its urban poor having unplanned/poor housing, insufficient unsafe water sources, unhygienic environments, low levels of education, high level of unemployment, and poor health, and nutrition indicators [22,23]. Although underweight poses significant health risks and is presumed to be more prevalent among lactating mothers in urban slums than in other areas, there is paucity of data on its prevalence and associated factors in the urban slums of Kampala City. This knowledge gap hinders the development of evidence-based interventions and informing policies to improve maternal and child health outcomes. Understanding the burden and correlates of maternal underweight in these vulnerable communities is important for designing targeted health strategies and for advancing optimal nutrition as part of Uganda’s efforts to lower undernutrition rates. Therefore, this study aimed to estimate the prevalence and determine the correlates of underweight among lactating mothers aged 20–49 years living in the urban slums of Kampala City.

## Methods

### Ethics statement

Ethical approval was obtained from the Makerere University School of Public Health Higher Degrees Research and Ethics Committee (MakSPH-REC 627/2025). Permission to carry out the study was obtained from Kampala City Authority (DPHE/KCCA/1302/01) and from the local council chairpersons of the ten zones where the data collection took place. Participation was voluntary, free from coercion, and participants reserved the right to withdraw from the study at any time. All experimental protocols were performed in accordance with the Declaration of Helsinki. Written informed consent was obtained from all study respondents, no names of participants were used, and all the information obtained was kept confidential. All lactating mothers identified to be underweight were counselled on optimum feeding during lactation and were linked to further nutrition care at various nutritional centers where necessary.

### Study area, design, period, and population

A community-based cross-sectional convergent mixed-methods study design employing both quantitative methods for data collection and qualitative approaches to examine participants’ perceptions was carried out from 06^th^ June 2025–06^th^ July 2025 in the urban slums of Kampala City in Uganda. Kampala, Uganda’s capital and largest city, is located in the central region and comprises five administrative divisions: Kampala Central, Kawempe, Makindye, Nakawa, and Rubaga [24]. Kampala has approximately 20 large urban slums including Katanga, Bwaise, Banda, Kisenyi, Kamwokya, Kibuli, Kasokoso, Nankulabye, Kinawataka, Katwe II, Naguru, Butabika, Kalwerwe, Makerere III, Kyebando, and Namuwongo among others [24]. The study population included lactating mothers aged 20–49 years breastfeeding children aged 6–23 months and living in Kampala urban slums. This selection criteria ensured mothers had completed postpartum recovery and their children had initiated complementary feeding, reducing potential confounders affecting maternal weight status [25]. This study focused on adult lactating mothers aged 20–49 years; therefore, mothers aged 15–19 years were not included in the study population. Additionally, key informant interviews (KIIs) were conducted with health workers responsible for supervising maternal and child health services across Kampala’s urban slum communities. These KII participants were selected because of their direct oversight of health facilities serving the targeted population, as most mothers living in these urban slums access health care through these supervised facilities.

### Sample size determination and sampling procedure

An adjusted sample size of 285 lactating mothers aged 20–49 years living in the urban slums of Kampala City was estimated using the Kish Leslie formula for estimating sample size in cross-sectional studies [26], while considering an expected prevalence of underweight among lactating mothers of 8% [5], a 5% level of significance, a design effect of 2, and a 20% potential non-response [27]. Multistage random sampling was used to select one slum from each of the five divisions of Kampala City, and two zones from each slum. A sampling frame of eligible lactating mothers aged 20–49 years in all the selected zones (10 zones) was developed with the help of the community health workers (CHWs) of the selected zones. Probability proportionate to size sampling was used to determine the number of eligible lactating mothers to be sampled per zone. The total number of eligible mothers across all the 10 selected zones was calculated. The study sample was then allocated proportionally to each zone based on the number of eligible mothers in that zone relative to the total number of eligible mothers across all the 10 selected zones. Random sampling was then used to select the eligible lactating mothers in each zone. All lactating mothers aged 20–49 years breastfeeding children aged 6–23 months and had resided in Kampala’s urban slums for three or more months were eligible for inclusion. Inclusion of mothers who had lived in the slums for at least three months was because these had experienced the environmental conditions in the slum for a significant amount of time. All eligible mothers aged 20–49 years who were very ill and physically unable to undergo anthropometric measurements were excluded from the study. Additionally, all eligible mothers who were pregnant, those who had missed their periods, and those who were not sure if they were pregnant or not were excluded from the study.

### Study variables

#### Outcome variable.

The outcome variable was maternal underweight status among lactating mothers, assessed using BMI calculated as weight in kilograms divided by height in meters squared (kg/m²). BMI was dichotomized as normal if the BMI was ≥ 18.5 kg/m and underweight if the BMI was < 18.5 kg/m^2^ [2].

#### Exposure variables.

The exposure variables explored through extensive literature review included dietary intake; maternal health status; household food security; maternal care and health services; water, sanitation, and hygiene (WASH); and sociodemographic characteristics [5,8,11,12,28–40]. The sociodemographic characteristics included mother’s age in completed years, religion, tribe, marital status, and type of marriage if married or cohabiting; household head; highest level of education attained by the lactating mother and her husband if applicable; employment status of the lactating mother and her husband if applicable; household size; age of the current child in completed months; and number of children under five years in the household. The dietary intake characteristics included number of times the lactating mother ate in 24 hours; extra meals given to the mother because she was breastfeeding; alcohol and tobacco use; and the minimum dietary diversity for women (MDD-W) measured using the Food and Agriculture Organization (FAO)’s guidelines [41]. MDD-W is a dichotomized indicator assessing whether women of reproductive age consumed foods from at least 5 out of 10 standardized food groups during the preceding 24-hour recall period. The household food security characteristics included the number of household members earning an income and household food insecurity status assessed using the household food insecurity access scale (HFIAS). HFIAS scores were dichotomized into food-secure households (scores 0–9) and food-insecure households (scores 10–27) based on validated cut-off points [42]. The WASH characteristics included the source of drinking water, drinking water intake, drinking water treatment, availability of a latrine or toilet, handwashing facility, waste disposal method, and daily domestic water access categorized as adequate or inadequate according to the WHO recommendation of at least 20 liters of safe water per person per day to meet essential health and hygiene needs [43].

The maternal care and health services characteristics included antenatal care (ANC) and postnatal care (PNC) attendances; number of ANC visits; nutrition counselling during ANC, and PNC; use of a contraceptive method, age at first pregnancy; delivery place; mode of delivery; planned/unplanned pregnancy; type of delivery; history of abortion; gravidity; parity; current birth interval; child exclusive breastfeeding history; and breastfeeding history in the last 24 hours. The maternal health status characteristics included maternal history of diseases or illnesses such as malaria, diarrhea, dysentery, acute respiratory tract infections, diabetes, hypertension, gestational diabetes, gestational hypertension, cancer, HIV (Human Immunodeficiency Virus), and sickle cell disease (SCD). Additional maternal health status characteristics included deworming history, iron and folic acid supplementation history during pregnancy, and maternal depression assessed using the 9‑item Patient Health Questionnaire (PHQ‑9), a validated screening instrument that measures depressive symptoms experienced during the preceding two weeks. Lactating mothers were categorized as having depression (PHQ‑9 scores of 10–27) or non‑depressed (PHQ‑9 scores of 0–9) based on established clinical cut-off points [44,45].

### Data collection methods and anthropometric assessment

Five research assistants administered a structured questionnaire among eligible lactating mothers using face-to-face interviews and collected quantitative data on dietary intake characteristics, maternal health status characteristics, household food security characteristics, maternal care and health services characteristics, WASH characteristics, socio-demographic characteristics, and anthropometric measurements. A key informant interview (KII) guide was used to explore perceptions of health workers on the factors associated with a higher and lower prevalence of underweight among lactating mothers living in the urban slums of Kampala City. Ten KIIs that lasted approximately 30–45 minutes each were conducted following the approved KII guide. The saturation point, where no new information was contributed and every case confirmed an already predicted norm; was taken into consideration [46,47]. All interviews were audio-recorded with participant consent and were supplemented by detailed interviewer field notes.

Anthropometric data were collected on height, and weight and BMIs were calculated and used to determine the prevalence of underweight among lactating mothers. Pre-tested and calibrated digital SECA 874 flat scales were used to measure weight according to WHO recommendations [48] by first placing the scale on a firm, flat surface. The scale was turned on and waited upon until the display showed 0.0. Mothers were then asked to remove their footwear (shoes, slippers, sandals) and socks, as well as any heavy belts, and to empty their pockets of items such as mobiles, wallets, and coins. They then stepped onto the scale with one foot on each side and were instructed to stand still, face forward, placing their arms at their sides, and waiting until asked to step off. The weight was then recorded to the nearest 0.1 Kg. This process was then repeated for a second weight measurement and the average weight was calculated. Three-in-one-piece wooden height boards approved by the United Nations Children’s Fund (UNICEF) were used to measure the height according to the WHO recommendations [48] by first separating the three pieces of the measuring board by unscrewing the knot at the back, then assembling them in the correct order and locking the latches. The board was then placed on a firm surface against a wall. The mothers were asked to remove footwear (shoes, slippers, sandals), headgear (hats, caps, hair bows, combs, ribbons), and to press down any fancy or high hairdos. If removing a scarf or veil would be insensitive, the measurement was taken over light fabric. Participants stood with feet together, heels against the backboard, knees straight, and looked straight ahead without tilting their head, ensuring their eyes were level with their ears. The measuring arm was gently lowered onto the head while the participant was asked to breathe in and stand tall. Height was read and measured to the nearest 0.1 meters, after which the participant stepped away from the board. The measurement was then recorded into the questionnaire. This process was then repeated for a second height measurement and the average height was calculated. BMI was then calculated by dividing a person’s weight in kilograms by their height in meters squared (kg/m^2^) [2].

### Data collection tools, quality assurance and control

Quantitative data collection was conducted by trained research assistants through face to face interviews using a structured electronic Kobo-Collect software questionnaire written in English with inbuilt guidance hints, skip logics, validation criteria, if statements, and mandatory fields that ensured all required questions were answered correctly, accurately, and high-quality data collection took place without any missing data. Physical copies of the translated consent forms and questionnaires in *Luganda* (the mostly spoken local language in Kampala City) were also provided to each research assistant daily.

Five research assistants with healthcare backgrounds at bachelor’s degree level and previous experience in quantitative research and electronic data collection were recruited. All the research assistants were fluent in English and *Luganda* and underwent a comprehensive training to familiarize them with the study objectives, sample selection, data collection tools, ethical considerations, approach to the respondent’s issues of privacy and confidentiality, research ethics, and administering informed consent to the respondents. The training also included practical sessions on administering electronic questionnaires, conducting interviews, and taking anthropometric measurements. Data collection tools were pre-tested among 15 eligible lactating mothers in Bwaise slum, a community excluded from the main study sample, to ensure tool validity and procedural reliability. The pretesting feedback was systematically incorporated to refine questionnaire clarity, interview guide effectiveness, and measurement procedures before field implementation. At the end of each day each research assistant cross-checked the completed forms for accuracy and consistency before submitting them to the server. The principal investigator supervised the data collection exercise by working with the research assistants while interviewing some of the study respondents.

### Data management and analysis

A password-protected Kobo Collect account owned by the principal investigator was used to collect the quantitative data and was accessible only to the principal investigator. Additionally, the final dataset was downloaded as an excel dataset and stored in a password protected drive. The audio files and soft copies of the transcripts that were generated from the KIIs were also stored in the password protected Google drive of the principal investigator, while the interviewer’s notes and hard copies of the generated transcripts from the KIIs were stored under lock and key in the principal investigator’s office cabinet.

Quantitative data analyses were performed using STATA/ SE v15 (Stata Corp LLC, Lakeway Drive, TX, USA). Descriptive statistics for key sociodemographic and maternal characteristics were summarized. The analyses in the study findings were conducted using a three-tiered approach: univariate, bivariable, and multivariable regression models. A prespecified primary multivariable modified Poisson regression model was fitted to determine the correlates of underweight among lactating mothers, guided by the United Nations Children’s Fund (UNICEF) conceptual framework on maternal and child nutrition [49] and previous literature [5,8,11,12,28–40]. Covariates included in the model were selected a priori based on conceptual relevance and prior evidence [5,8,11,12,28–40,49] rather than statistical significance. The model included underweight as the outcome and seven exposure variables representing immediate causes (MDD–W, meal frequency, depression, and deworming history) and underlying determinants (HFIAS, household size, and maternal employment status) of maternal underweight [49]. To account for the multistage sampling design and improve the representativeness of the study estimates, sampling weights were calculated as an inverse probability of selection based on the zone level sampling frames and incorporated into all analyses [50,51]. Because underweight was assessed as a binary outcome and its prevalence (13.7%) was more than 10% (not rare), sampling weighted modified Poisson regression analysis with cluster robust standard errors, via generalized linear models with family (Poisson) and link (log) was used to estimate prevalence ratios (PRs) with corresponding 95% confidence intervals (CIs) [52,53]. This approach was used because odds ratios tend to overestimate the strength of association when outcomes are not rare [54,55]. To account for potential correlation among observations within zones arising from the multistage sampling design, cluster robust standard errors were estimated at the zone level [56]. Bivariable associations generating crude PRs (cPRs) and corresponding 95% CIs were examined descriptively for variables included in the primary model. Covariates with *p* < 0.05 were considered correlates of underweight among lactating mothers aged 20–49 years living in Kampala’s urban slums.

Sensitivity analyses were conducted to assess the robustness of the prespecified primary multivariable Poisson regression model by sequentially refitting the model with additional, conceptually distinct covariate blocks one at a time. This block‑based approach follows methodological guidance that emphasizes principled, theory‑driven sensitivity analyses rather than exhaustive model enumeration [57–60]. Three sensitivity blocks were specified a priori and added sequentially: a reproductive covariate block (maternal age, and number of children born alive), a socioeconomic covariate block (mother’s highest education, and household income earners), and a marital structure block (marriage type). Only estimates from the primary model variables were displayed in sensitivity forest plots to facilitate assessment of effect‑estimate stability across alternative model specifications.

KII audio recordings were transcribed verbatim, and interviewer field notes were reviewed to provide contextual understanding and supplement the transcribed data. Data were manually coded/themed by the research team with the support of excel tables. Cleaning of themes was completed by the principal investigator and reviewed by the research team. Key issues, perspectives, and recurring themes from each question set were identified and organized into categories to answer the research question using inductive thematic content analysis. When discrepancies in analysis occurred among the researchers, they were resolved as a team during coding/sub-theming and the thematic analysis. However, due to the nature of the participants’ responses, there was minimal disagreement among researchers during the data analysis. Interpretation of the results was presented in the discussion and included triangulation of the quantitative and qualitative findings for comprehensive and better contextual understanding of findings.

## Results

### Background characteristics of the study population

A total of 285 lactating mothers aged 20–49 years living from Kampala’s urban slums were recruited into the study. Complete data was obtained from all participants achieving a 100% response rate and ensuring the final analysis was based on the calculated sample size.

#### Sociodemographic characteristics of the respondents.

Almost one-third (34.7%) of the study participants were 25–29 years old. The mean ± SD maternal age was 29 ± 5, with participants ranging from 20 to 41 years of age. Among the 285 lactating mothers that were interviewed, majority (77.5%) had attained secondary education, almost half (48.8%) were stay home mothers, while three-quarters (75.8%) belonged to households headed by their husbands (Table 1).

**Table 1:** Sociodemographic characteristics of the respondents.

Characteristic	Min	Max	Mean(±SD)	Frequency(n = 285)	Percentage
**Mother’s age**	20	41	29(5)		
20–24				52	18.3
25–29				99	34.7
30–34				76	26.7
35–39				50	17.5
40–44				8	2.8
**Mother’s highest education**					
Diploma and more				18	6.3
Primary				46	16.1
Secondary				221	77.6
**Mother’s employment status**					
Employed				146	51.2
Stay home mothers				139	48.8
**Household head**					
Husband				216	75.8
Lactating mother				57	20.0
Others				12	4.2
**Marital status**					
Married/cohabiting				218	76.5
Divorced/separated				42	14.7
Others				25	8.8
**Marriage type** ^**†**^					
Monogamy				173	79.3
Not sure				13	6.0
Polygamy				32	14.7
**Husband’s occupation** ^**†**^					
Businessman				121	55.5
Transport sector				74	33.9
Others				23	10.6
**Husband’s highest education** ^**†**^					
Diploma and more				65	29.8
Primary				15	6.9
Secondary				138	63.3
**Husband’s employment** ^**†**^					
Employed				218	100.0
**Household size**	3	9	5(1)		
≥5				204	71.6
<5				81	28.4
**Children in the household**					
One	1	3	1(1)	212	74.4
Two–Three				73	25.6
**Child age (months)**	6	21	10(3)		
6–11				201	70.5
12–17				75	26.3
18–23				9	3.2

^†^Among 218 lactating mothers who were married/cohabiting.

Abbreviations: Min: minimum, Max: Maximum, SD: Standard deviation

A total of 218 (76.5%) were married/cohabiting of which 173 (79.3%) were in a known monogamous marriage, 121(55.5%) had husbands who were businessmen, 138(63.3%) had husbands who had attained a secondary education, and all 218(100%) had husbands who were employed. Out of all the lactating mothers recruited in the study, 204 (71.6%) belonged to households having five or more members with a minimum of three, a maximum of nine and a mean of 5(±1) household members. A total of 212 (74.4%) lactating mothers lived with one child, with a minimum of one, maximum of three, and a mean of 1(±1) children under five years of age. Most of the lactating mothers (70.5%) had their youngest children aged 6–11 months with ages ranging from 6–21 months, and a mean 10(±3) months (Table 1).

#### Dietary intake, household food security, and WASH characteristics of the respondents.

Most of the lactating mothers (77.9%) lived in households that ate three or less meals a day with the household meal frequency ranging from two to six meals per day, and a mean of 3(±1) meals per day (Table 2).

**Table 2:** Dietary intake, household food security and WASH characteristics.

Characteristic	Min	Max	Mean(±SD)	Frequency (n = 285)	Percentage
**Meal frequency in a day**	2	6	3(1)		
≤3				222	77.9
>3				63	22.1
**MDD–W**	2	7	5(1)		
No				74	26.0
Yes				211	74.0
**Extra meals during lactation**					
No				184	64.6
Yes				101	35.4
**Frequency of extra meals**	0	3	1(1)		
0–1				199	64.6
2–3				86	35.4
**Alcohol consumption**					
No				275	96.5
Yes				10	3.5
**Cigarette smoking**					
No				285	100.0
**HFIAS**	0	18	3(3)		
Food secure				183	64.2
Food insecure				102	35.8
**Household income earners**	1	3	1(1)		
One				196	68.8
Two-Three				89	31.2
**Drinking water source**					
Tap				280	98.2
Well				5	1.8
**Daily water intake (liters)**	0.5	3.5	2.2(0.7)		
<2.7				147	51.6
≥2.7				138	48.4
**Drinking water treatment method**					
Boiling				284	99.7
None				1	0.3
**Availability of toilet facility at home**					
No				1	0.3
Yes				284	99.7
**Availability of hand washing facility**					
No				79	27.7
Yes				206	72.3
**Household waste disposal**					
Burning				41	14.4
KCCA waste collectors				237	83.1
Pit				7	2.5
**Daily domestic water access(liters)**	20	140	65(18)		
Inadequate				255	89.5
Adequate				30	10.5

Abbreviations; Min: minimum, Max: Maximum, SD: Standard deviation, MDD–W: minimum dietary diversity for women, HFIAS: household food insecurity access sale, KCCA: Kampala Capital City Authority.

Almost two thirds of the lactating mothers (64.6%) did not attain the MDD-W with an individual dietary diversity score (IDDS) ranging from two to seven with a mean of 5(±1). Slightly more than a third of the lactating mothers (35.4%) received extra meals during lactation, with the extra meal frequency ranging from 0 to three meals and a mean of 1(±1) extra meals per day. Almost two thirds of the lactating mothers (64.2%) lived in food insecure households with HFIAS scores ranging from 0–18 and a mean score of 3(±3). Slightly more than two thirds of the lactating mothers (68.8%) lived in households with only one income earner with the number of household income earners ranging from one to three and a mean of 1(±1). Most of the lactating mothers (51.6%) drunk less than 2.7 liters of water a day with daily water intake ranging from 0.5 to 3.5 liters and a mean of 2.2(±0.7) liters. Most of the lactating mothers (89.5%) belonged to households that had inadequate daily domestic water access with daily domestic water access ranging from 20 to 140 liters and a mean of 65(±18) liters. Among all the lactating mothers recruited in the study, 10 (3.5%) consumed alcohol on a daily basis, none of them (100%) smoked cigarettes, 280 (98.2%) obtained their drinking water from a tap (pipped water), almost all (99.3%) boiled their drinking water and had toilet facilities at home, 206 (72.3%) had hand washing facilities, and 237 (83.1%) disposed off their domestic waste through the KCCA waste collection services (Table 2).

#### Maternal care and health services characteristics.

All the lactating mothers (100%) in this study had attended at least two ANC session during their most recent pregnancy with most of them attending ANC for 5–8 times with ANC attendances ranging from two to eight and a mean of 5(±1) ANC attendances (Table 3).

**Table 3:** Maternal care and health services characteristics.

Characteristic	Min	Max	Mean(±SD)	Frequency (n = 285)	Percentage
**Ante natal clinic (ANC) Attendance**					
Yes				285	100.0
**Frequency of ANC visits**	2	8	5(1)		
2–4				85	29.8
5–8				200	70.2
**Nutrition counselling during ANC**					
No				4	1.4
Yes				281	98.6
**Intended to have youngest child**					
Yes				228	80.0
No				57	20.0
**Delivery place**					
Health facility				283	99.3
Traditional birth attendant				2	0.7
**Mode of delivery**					
Cesarean section				109	38.2
Normal vaginal				176	61.8
**Birth type**					
Singleton				273	95.8
Twins				12	4.2
**Number of pregnancies**	1	8	3(1)		
1–2				98	34.4
≥3				187	65.6
**Number of children born alive**					
Multipara				199	69.8
Primipara	1	7	2(1)	86	30.2
**Abortion history**					
No				195	68.4
Yes				90	31.6
**Current birth interval** ^‡^					
≥ 2 years				209	82.6
< 2 years				44	17.4
^**†**^ **Current contraceptive use**					
Yes				112	39.3
No				173	60.7
**Age at first pregnancy**	15	29	21(2)		
15–17				24	8.4
18–19				30	10.5
20–24				212	74.4
25–29				19	6.7
**Post-natal clinic (PNC) attendance**					
Yes				257	90.2
No				28	9.8
**Nutrition counselling during PNC** ^**§**^					
Yes				256	99.6
No				1	0.4
**Exclusively breastfed child**					
Yes				249	87.4
No				36	12.6
**Breastfed the child in last 24 hours**					
Yes				281	98.6
				4	1.4
**Breastfeeding frequency in last 24 hours** ^**¶**^	3	12	8(3)		
3-5				50	17.8
≥6				231	82.2

^‡^Among 253 mothers who had at least 1 previous live birth, ^§^Among 257 mothers who attended PNC, ^¶^Among 281 mothers who breastfed their children in the last 24 hours.

Abbreviations: Min: minimum, Max: Maximum, SD: Standard deviation.

Most of the lactating mothers (98.6%) received nutrition counselling during their ANC visits, 228 (80%) intended to have their youngest child, 283 (99.3%) delivered from health facilities, 176 (61.8%) had spontaneous vaginal deliveries, 273 (95.8%) had singleton babies, 195 (68.4%) had no abortion history, and 209 (82.6%) of those with more than one previous pregnancy had an interval of less than 2 years between their last two pregnancies. Two thirds of the lactating mothers (65.6%) had more than two pregnancies before with a range of one to eight pregnancies and mean of 3(±1) pregnancy. A majority of the lactating mothers (69.8%) were multiparous with a range of one to seven previous live births and a mean parity of 2(±1). One hundred twelve (39.3%) of the lactating mothers were using a contraceptive method. Three quarters of the lactating mothers had their first pregnancy at ages between 20–24 years with age at first pregnancy ranging from 15 to 29 years and a mean of 21(±2) years. Most of the lactating mothers (90.2%) attended PNC of which 256 (99.6%) received nutrition counselling. A majority of the lactating mothers (87.4%) reported having exclusively breastfed their youngest child, and 281 (98.6%) had breastfed their child in the 24 hours before the study. Of those who had breastfed their children in the previous 24 hours, 231 (82.2%) had breastfed their children more than five times, with breastfeeding frequency ranging from three to 12 times and a mean of 8(±3) (Table 3).

#### Maternal health status characteristics.

Among the study participants, almost a quarter (23.5%) had a history of a positive malaria diagnosis, the majority (68.4%) reported having an acute respiratory tract infection in the two weeks preceding the study, and a few (9.8%) had recently experienced a diarrheal episode (Table 4).

**Table 4:** Maternal health status characteristics.

Characteristic	Frequency(n = 285)	Percentage
**Malaria in the last 2 weeks**		
No	218	76.5
Yes	67	23.5
**Diarrhea in the last 2 weeks**		
No	257	90.2
Yes	28	9.8
**Acute RTI in the last 2 weeks**		
No	90	31.6
Yes	195	68.4
**Gestational Diabetes Mellitus**		
No	285	100.0
**Gestational hypertension**		
No	211	74.0
Yes	74	26.0
**Diabetes Mellitus**		
No	281	98.6
Yes	4	1.4
**Hypertension**		
No	260	91.2
Yes	25	8.8
**Cancer**		
No	285	100.0
**HIV**		
No	234	82.1
Yes	51	17.9
**Sickle cell disease**		
No	283	99.3
Yes	2	0.7
**Depression**		
No	208	73.0
Yes	77	27.0
**Deworming in the last 6 months**		
No	103	36.1
Yes	182	63.9
**Iron/folic acid supplementation during pregnancy**		
Yes	285	100.0

Abbreviation; RTI: Respiratory tract infection, HIV: Human Immunodeficiency Virus.

During their last pregnancy, all the lactating mothers (100%) were not affected by gestation diabetes mellitus (GDM), while 74 (26.0%) were affected by gestational hypertension. Of all the lactating mothers recruited in this study, 4 (1.4%) were living with chronic diabetes mellitus, 25 (8.8%) were living with chronic hypertension, none was living with cancer, 51 (17.9%) were living with HIV, and 2 (0.7%) were living with sickle cell disease. Slightly more than a quarter of the lactating women (27.0%) reported living with depression, 182 (63.9%) had taken deworming tablets in the six months preceding the study, and all of the lactating women had taken iron/folic acid supplementation during their most recent pregnancy (Table 4).

### Prevalence of underweight among lactating mothers aged 20–49 years living in the urban slums of Kampala City

The prevalence of underweight among lactating mothers aged 20–49 years with children aged 6–23 months living in Kampala’s urban slums was 13.7% (95% CI: 9.9%–18.2%).

### Correlates of underweight among lactating mothers aged 20–49 years living in the urban slums of Kampala City

Results in Table 5 showed that household food insecurity, household size of fewer than five members, daily household meal frequency of more than three meals, and attaining minimum dietary diversity for women (MDD-W) were associated with underweight among lactating mothers aged 20–49 years living in the Kampala urban slums.

**Table 5:** Correlates of underweight among lactating mothers aged 20–49 years living in the urban slums of Kampala City.

Characteristic	Underweight (n = 285)		Crude PR (95% CI)	Adjusted^†^ PR (95% CI)	*p-*value
	No	Yes			
**HFIAS**					
Food secure	183	0	1.0	1.0	
Food insecure	63	39	1.4 (1.3–1.5)	1.4 (1.2–1.6)	<0.001***
**Deworming in last 6 months**					
No	71	32	1.0	1.0	
Yes	175	7	0.79 (0.75–0.84)	0.99 (0.87–1.1)	0.897
**Household size**					
≥5	167	37	1.0	1.0	
<5	79	2	0.87 (0.81–0.93)	0.94 (0.89–0.99)	0.023*
**Depression**					
No	191	17	1.0	1.0	
Yes	55	22	1.2 (1.1–1.3)	0.96 (0.84–1.1)	0.566
**Meal frequency in a day**					
≤3	183	39	1.0	1.0	
>3	63	0	0.85 (0.79–0.92)	0.74 (0.66–0.83)	<0.001***
**Mother’s employment status**					
Employed	114	32	1.0	1.0	
Stay home mothers	132	7	0.86 (0.81–0.92)	0.98 (0.93–1.03)	0.414
**MDD–W**					
No	46	28	1.0	1.0	
Yes	200	11	0.76 (0.69–0.84)	0.91 (0.84–0.96)	0.026*

* Significant association at *p* < 0.05; ** Significant association at *p* < 0.01; *** Significant association at *p* < 0.001.

The prevalence of underweight among lactating mothers who lived in food insecure households was 40% more than that among mothers who lived in food secure households (aPR: 1.4, CI: 1.2–1.6, *p* < 0.001)**.** Conversely, the prevalence of underweight among lactating mothers who lived in households with less than five household members were 6% lower than that among mothers who lived in households with five or more members (aPR: 0.94, CI: 0.89–0.99, *p* = 0.023). The prevalence of underweight among lactating mothers who lived in households that ate more than three meals a day was 26% lower than that among mothers who lived in households that ate 2–3 meals a day (aPR: 0.74, CI: 0.66–0.83, *p* < 0.001). Similarly, the prevalence of underweight among lactating mothers who attained the recommended minimum dietary diversity for women (MDD-W) was 9% lower than that among mothers who did not attain the recommended MDD-W (aPR: 0.91, CI: 0.84–0.96, *p* = 0.026).

### Sensitivity analyses

Across all the three sensitivity analyses, associations observed in the primary multivariable regression model remained directionally consistent and of similar magnitude, supporting the robustness of the findings. Forest plots show that the effect estimates for variables in the primary model were stable after additional adjustment for the reproductive covariate block (maternal age, and number of children born alive; Fig 1), the socioeconomic covariate block (mother’s highest education, and household income earners; Fig 2), and the marital structure block (marriage type; Fig 3).

**Fig 1:**
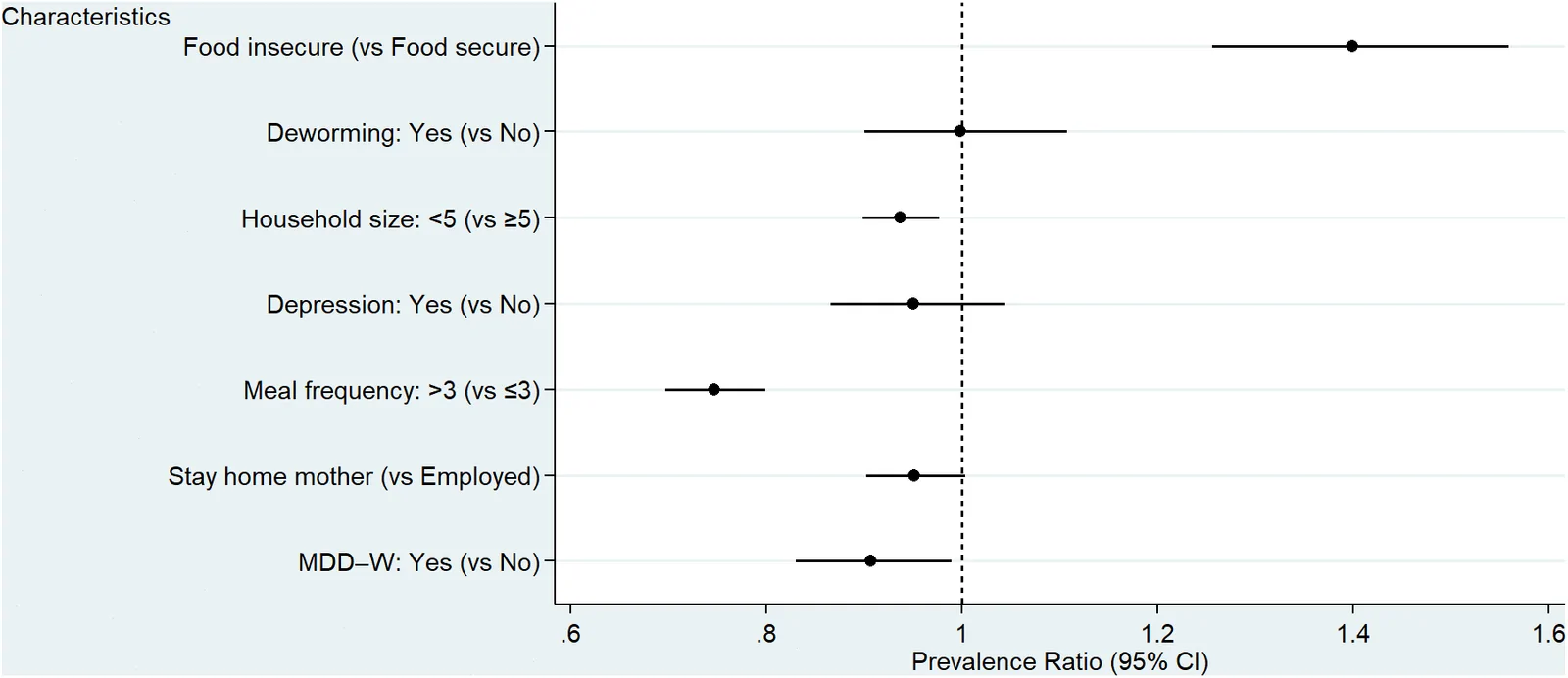
Sensitivity analysis demonstrating robustness of primary model estimates after adjusting for the reproductive factors among lactating mothers. Forest plot showing adjusted prevalence ratios (PRs) and 95% confidence intervals (CIs) for variables in the sensitivity model that additionally adjusted for reproductive factors (maternal age and number of children born alive). Only estimates for the primary model variables are displayed to assess the stability of associations across model specifications.

**Fig 2:**
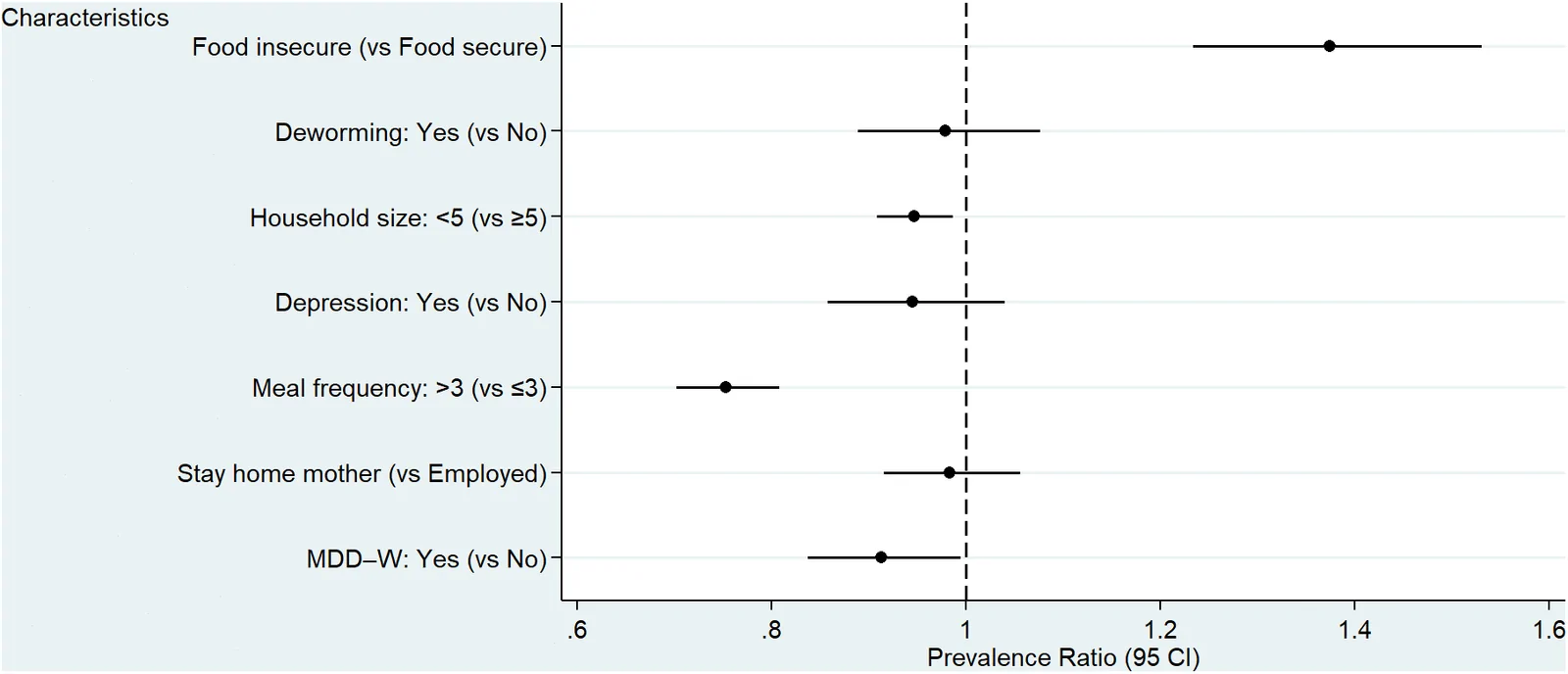
Sensitivity analysis demonstrating robustness of primary model estimates after adjusting for the socioeconomic factors among lactating mothers. Forest plot presenting adjusted prevalence ratios (PRs) and 95% confidence intervals (CIs) for variables in the sensitivity model that additionally adjusted for socioeconomic factors (mother’s highest education and household income earners). Estimates are shown to facilitate comparison of effect magnitude and direction across models.

**Fig 3:**
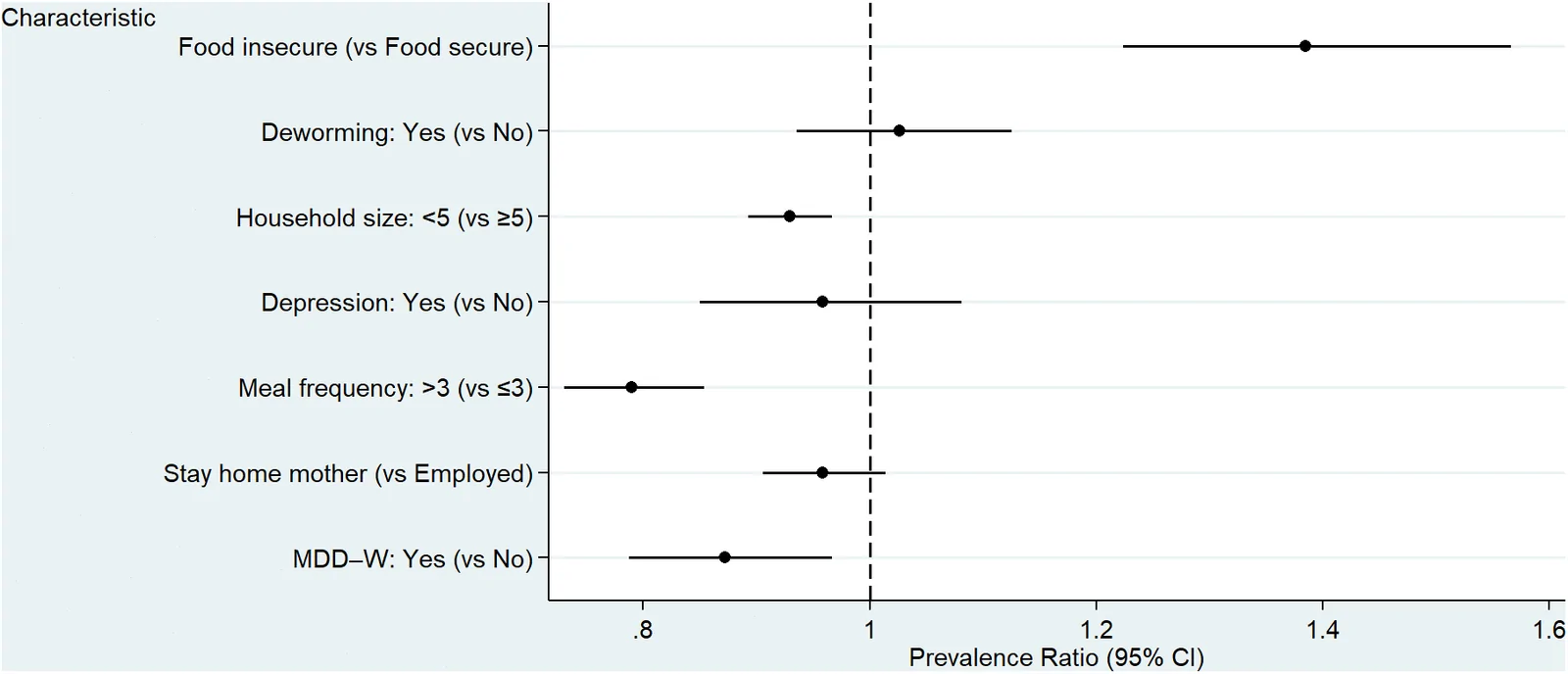
Sensitivity analysis demonstrating robustness of primary model estimates after adjusting for marital structure among lactating mothers. Forest plot displaying adjusted prevalence ratios (PRs) and 95% confidence intervals (CIs) for variables in the sensitivity model that additionally adjusted for marital structure (marriage type). Only primary model estimates are shown to evaluate robustness of associations under alternative model specifications.

### Perceptions of health workers on the factors associated with a higher and lower prevalence of underweight among lactating mothers living in the urban slums of Kampala City

Ten KIIs among key stakeholders that included medical doctors and senior nursing officers were conducted. Of the 10 KIIs, 6 (60.0%) were female and the remaining 4 (40.0%) were males. Out of the 10 KIIs, 5 (50.0%) were Division Medical Officers (DMVs) of the five divisions in Kampala City and the remaining 5 (50.0%) were division in charges of maternal and child health services of the five divisions of Kampala City. Results from the KIIs on the perceptions of the health workers on the factors associated with a higher and lower prevalence of underweight among lactating mothers living in the urban slums of Kampala City were divided into five major emerging themes which included: perceptions on the current state of underweight among lactating mothers in the urban slums of Kampala City; maternal related factors perceived to be associated with a lower prevalence of underweight among lactating mothers; family and societal related factors perceived to be associated with a lower prevalence of underweight among lactating mothers; maternal related factors perceived to be associated with a higher prevalence of underweight among lactating mothers; and family and societal related factors perceived to be associated with a higher prevalence of underweight among lactating mothers.

#### The current state of underweight among lactating mothers in the urban slums of Kampala City.

Most key informants noted that the prevalence of underweight among lactating mothers living in the urban slums of Kampala City was notably high and exceeded the national average. They emphasized that these mothers faced multiple, interrelated challenges that negatively affected their nutritional status, and made it difficult for them to maintain a healthy weight during the lactation period.

*“The prevalence of underweight among lactating mothers in the urban slums of Kampala City is higher than the national average. These mothers face a complex range of challenges that hinder them from achieving adequate nutritional status.”* (KII-08, Makindye Division Headquarters)

#### Maternal related factors perceived to be associated with a lower prevalence of underweight among lactating mothers.

One of the perceived facilitators mentioned by the key informants was eating frequent meals. They noted that eating frequent meals facilitated a good nutrition and normal weight status among lactating mothers. They noted that the more meals the lactating mother consumes, the greater the chances of consuming a variety of nutritious foods that meet the increased calorie and nutrition needs of the lactating mothers for a healthy life and sufficient quality breastmilk production.

*“I also believe that meal frequency is an important facilitator of normal nutritional status among lactating mothers. The more meals a lactating mother consumes, the greater the likelihood of meeting her increased calorie and nutrient needs for maintaining good health and producing sufficient, high-quality breastmilk.”* (KII-04, Kawempe Division Headquarters)

High educational attainment among lactating mothers was also identified by the key informants as a facilitator of good nutritional status. They noted that mothers who completed higher levels of education often gained knowledge about maternal nutrition during pregnancy and lactation, which helped them make better dietary choices. They also mentioned that such mothers were more likely to secure higher-paying jobs, which provided them with enough money to purchase the necessary foods to maintain a good nutritional status.

*“I also think that lactating mothers who have attained higher levels of education are less likely to be underweight. This could be because, at higher levels of education, they often gain knowledge about maternal nutrition during pregnancy and lactation, which helps them make better dietary choices. Additionally, mothers with higher education are more likely to have better-paying jobs compared to those with no formal education. This allows them to have enough money to buy all the foods they need to achieve a good nutrition status.”* (KII-05, Nakawa Division Headquarters)

The key informants emphasized that achieving the minimum dietary diversity for women played a crucial role in promoting good nutritional status and maintaining a healthy weight among lactating mothers. They pointed out that although few lactating mothers were aware of the importance of meeting the dietary standard, some managed to achieve it unintentionally through varied diets. According to the informants, consuming a wide range of food groups helped ensure that lactating mothers received the essential nutrients required to meet their increased dietary needs and to produce sufficient and nutritious breastmilk.

*“Few lactating mothers are aware of the importance of meeting the minimum dietary diversity for women, though some achieve it unknowingly. Minimum dietary diversity is a key facilitator of good nutrition and a healthy weight status among lactating mothers, as diverse diets provide a variety of foods rich in the nutrients essential for meeting daily dietary needs and supporting breastmilk production.”* (KII-01, Rubaga Division Headquarters)

It was also mentioned by the key informants that being employed and working away from home were barriers to achieving a healthy weight and good nutritional status among lactating mothers, while staying at home was seen as a facilitator. They explained that working mothers, after dedicating time to their jobs and caring for their husbands and children, often had little or no time to care for themselves. In contrast, they noted that housewives were able to use the time they would have spent working to focus on their own well-being.

*“I also believe that maternal unemployment may, in some cases, be a blessing in disguise for stay home mothers, as it can serve as a facilitator of good nutrition and healthy weight status among lactating mothers. This may be because housewives often have more time for self-care after attending to their husbands and other family members, unlike employed mothers who, after fulfilling work responsibilities and caring for their families, are often left with little or no time to focus on their own well-being.”* (KII-10, Kampala Central Division Headquarters)

#### Family and societal related factors perceived to be associated with a lower prevalence of underweight among lactating mothers.

The key informants noted that access to quality health services, including antenatal and postnatal care, was a key facilitator of normal nutritional status among lactating mothers. They explained that mothers who attended all recommended antenatal and postnatal visits received valuable counselling, including on maternal nutrition during pregnancy and lactation. This knowledge helped them understand what foods to eat and how frequently, which improved the quality of their dietary intake and supported better nutritional outcomes.

*“It’s our duty to provide quality health services to our people. When the lactating mothers have access to quality health services, including antenatal care and postnatal care, and they attend all the recommended visits, they can receive valuable nutrition counselling during pregnancy and lactation. This helps them understand what foods to eat and how often, which improves the quality of their dietary intake and promotes healthy weight outcomes.”* (KII-02, Rubaga Division Headquarters)

The KII findings also indicated that having fewer household members was a facilitator of healthy nutritional status among lactating mothers. They noted that smaller household sizes reduce competition for food, whereas larger households increase competition, often leaving lactating mothers with less food since they typically serve themselves last after everyone else has eaten.

*“Another factor that facilitates a normal nutritional status among lactating mothers, particularly in households within urban slums, is having a small household size. Fewer household members reduce competition for food and other resources, allowing lactating mothers greater access to adequate meals. This enables them to meet their dietary needs, maintain a healthy nutritional status, and produce sufficient, nutritious breastmilk. Larger household sizes increase competition for food, limiting access for lactating mothers. Since they often prioritize caring for other household members, they tend to eat last and may receive less food, which predisposes them to underweight”* (KII-06, Nakawa Division Headquarters)

#### Maternal related factors perceived to be associated with a higher prevalence of underweight among lactating mothers.

The KIIs also noted that short birth intervals were a barrier to achieving normal nutritional status among lactating mothers. They explained that closely spaced pregnancies can lead to nutrient depletion, making it difficult for mothers to maintain a healthy weight while breastfeeding. They noted that each pregnancy draws from the mother’s nutrient reserves, and when conception occurs before the reserves are replenished, it increases their risk of becoming underweight.

*“I also think that short birth intervals can negatively affect a lactating mother’s nutritional status. Every pregnancy depletes a woman’s nutrient stores, and when she conceives again before fully recovering, it becomes difficult for her to maintain a healthy weight while breastfeeding. That’s why many mothers who have multiple deliveries in a short period are often underweight and unhealthy*.” (KII-07, Makindye Division Headquarters)

The KII findings also revealed that chronic illnesses such as Sickle Cell Disease, and HIV/AIDS were significant barriers to achieving a healthy weight and good nutritional status among lactating mothers. They explained that these conditions often reduce food intake due to symptoms like nausea, vomiting, or medication side effects that cause appetite loss. Additionally, they also noted that those illnesses can impair the absorption of nutrients through gastrointestinal symptoms like diarrhea and gastritis and increase the body’s nutrient demands because of heightened metabolic activity, all of which contribute to undernutrition.

*“Maternal chronic diseases such as Sickle Cell Disease, and HIV/AIDS can prevent lactating mothers from achieving a healthy weight and good nutritional status. These conditions may reduce food intake due to symptoms like nausea and vomiting or side effects of medications that cause loss of appetite. They can also impair nutrient absorption through symptoms such as diarrhea and gastritis, and increase the body’s nutrient requirements due to elevated metabolic demands.”* (KII-09, Kampala Central Division Headquarters)

Recent abortion history was also identified by the key informants as one of the barriers to achieving normal nutritional status among lactating mothers. They mentioned that complications from abortion, such as anemia and infections like septicemia, can affect the intake and utilization of nutrients by the lactating mother, leading to underweight.

*“I personally think that having a recent abortion history is one of the barriers to achieving a normal nutritional status among lactating mothers. Abortions are usually complicated by anemia and infections like septicemia, which affect the intake and utilization of nutrients by the lactating mother, thereby predisposing her to underweight.”* (KII-03, Kawempe Division Headquarters)

#### Family and societal related factors perceived to be associated with a higher prevalence of underweight among lactating mothers.

The KII findings also revealed that household food insecurity was a major barrier to achieving a healthy nutritional status among lactating mothers in the urban slums of Kampala City. They noted that many households in these areas lack land to grow food and rely entirely on purchasing it. They mentioned that due to unemployment or low income, most families cannot afford adequate food, resulting in insufficient intake for lactating mothers and increasing their risk of becoming underweight.

*“Household food insecurity is a major barrier to achieving adequate nutrition among lactating mothers in the urban slums of Kampala City. In these areas, residents typically lack space to grow their own food, making them entirely dependent on purchasing it. However, many have no employment or earn very low incomes, and the limited money they have is often insufficient to buy enough food for all household members. As a result, lactating mothers, like other members of the household, do not consume enough food, which puts them at risk of underweight.”* (KII-06, Nakawa Division Headquarters)

The key informants also mentioned that having polygamous husbands was a barrier to normal nutritional status among lactating mothers. They noted that many women depend on their husbands for food, and in polygamous settings, the husband’s resources are divided among multiple households, which reduces the financial support each mother receives, increasing the risk of food insecurity and predisposing lactating mothers to underweight.

*“…., additionally, lactating mothers with polygamous husbands are more likely to be underweight. Many of these mothers rely on their husbands for food, but in polygamous households, the husband’s resources are divided among multiple families. This reduces the financial support each woman receives, increasing the risk of food insecurity. On top of that, the competition and tensions with co-wives can cause psychological stress, which further contributes to poor nutritional status of the lactating mothers.”* (KII-03, Kawempe Division Headquarters)

## Discussion

This cross-sectional study conducted in the community examined the prevalence and correlates of underweight among lactating mothers aged 20–49 years living in Kampala’s urban slums. The prevalence of underweight among lactating mothers was 13.7%. Household food insecurity, household size of fewer than five members, daily household meal frequency of more than three meals, and attainment of MDD-W were the independent correlates of underweight among lactating mothers aged 20–49 years living in Kampala’s urban slums. Factors perceived to be associated with a higher prevalence of underweight included household food insecurity, and working mothers. Factors perceived to be associated with a lower prevalence of underweight included higher meal frequency, meeting the MDD‑W, stay home mothers, and fewer household members. Targeted interventions addressing these correlates could improve the nutritional well-being of lactating mothers in urban slum settings. This evidence supports better health outcomes for lactating mothers and their infants, contributing to the achievement of the sustainable development goals (SDGs) especially SDG 2 (Zero Hunger) and SDG 3 (Good Health and Well-Being), which emphasize improving maternal and child nutrition [61].

The prevalence of underweight among lactating mothers in this study was moderate based on WHO criteria, which classifies an underweight prevalence among lactating mothers of 5–9% as low (serving as a warning sign requiring monitoring), 10–19% as moderate (indicating a poor situation), 20–39% as high (indicating a serious situation), and ≥40% as very high (indicating a critical situation) [62]. The prevalence of underweight in the current study is higher than the national average of 8.2% among lactating mothers aged 20–49 years in Uganda [5]. This difference may be due to the fact that the national prevalence represents an average across all districts and includes lactating mothers of all ages and socioeconomic backgrounds, whereas the current study focused specifically on urban slums in largest city in the country. It is possible that women in such communities face unique and context specific challenges associated with low socioeconomic status [22,23]. Additionally, the prevalence of underweight among lactating mothers in the current study is higher than the national average of 9% among women of reproductive age (15–49 years) in Uganda [4]. This difference may be attributed to the fact that the current study focused solely on lactating mothers aged 20–49 years residing in low socioeconomic settings within the urban slums of Kampala City [19–21], while the national average includes all women of reproductive age, including pregnant, non-pregnant, and lactating women in Uganda. The prevalence of underweight among lactating mothers in this study is similar to the 13.5% reported in a study conducted in Northwest Ethiopia [29]. These findings are consistent with results from the KIIs, which indicated that the prevalence of underweight among lactating mothers in the urban slums of Kampala City was high and surpassed the national average of Uganda. On the contrary, the prevalence of underweight among lactating mothers in the current study was lower than the prevalence of underweight of 26.9% [28], 32.8% [36], 22.6% [30] and 25.9% [31], 21.2% [35] in Ethiopia. The difference could be attributed to several factors, including variations in study designs, seasonal changes, sampling methods, geographic locations, and socioeconomic factors between those studies and the current study. This unacceptable prevalence of maternal underweight based on the findings in this study highlights a significant public health problem and underscores the need for targeted interventions to improve nutrition status of lactating mothers living in the urban slums of Kampala City and other low socioeconomic settings.

In this study, household food insecurity was significantly associated with a higher prevalence of underweight among lactating mothers. The WHO and the Centers for Disease Control and Prevention (CDC) recommend lactating mothers to consume an adequate intake of nutritious foods to meet their own health needs and to produce sufficient, high-quality breastmilk [1,2,63]. In food-insecure households, access to sufficient, safe, and nutritious food is limited for all members, including the lactating mothers [1,2,63]. The limited access negatively affects their dietary intake, increasing their risk to underweight [39,40]. Additionally, in food insecure households, it is common for mothers to prioritize feeding their husbands and children first, leaving very little food for themselves which further increases their risk to underweight [39,40]. Our findings are similar to several other previous studies that also reported that household food insecurity was a significant risk factor of underweight among lactating mothers [11,28–30]. Additionally, the qualitative interviews revealed that household food insecurity was a barrier to achieving a healthy nutrition status among lactating mothers. Some of the KIIs suggested that the lack of food in the household lowered maternal dietary intake, leading underweight. Therefore, community sensitization on ways of achieving household food security such as backyard farming could help improve the nutrition status of lactating mothers in the urban slums of Kampala City and other low socioeconomic status settings.

Our study showed that lactating mothers living in households with less than five members were significantly less likely to be underweight. This may be because smaller household size is associated with lower competition for food and household resources, allowing mothers to better meet their own nutritional needs rather than prioritizing other family members [39,40]. This was consistent with reports from previous studies conducted in Ethiopia [8,31], and in China [32] which noted that small family size significantly decreased the risk of underweight among lactating mothers. The KII findings also indicated that having fewer household members was a facilitator of healthy nutritional status among lactating mothers. They noted that smaller household sizes reduce competition for food, whereas larger households increase competition, often leaving lactating mothers with less food since they typically serve themselves last after everyone else has eaten. So, community sensitization on the use of contraceptive methods as a way of reducing large family sizes and improved access to these methods in all health facilities could help improve the nutrition status of lactating mothers in the urban slums of Kampala City and other low socioeconomic status settings.

In this study, a daily meal frequency of more than three among the participants was significantly associated with a lower prevalence of underweight. Higher meal frequency likely improves both the quantity and quality of nutrient intake, helping to meet the increased calorie and nutrient demands of lactation, thereby supporting healthy maternal weight and adequate breastmilk production. These findings align with findings from several other studies conducted in Ethiopia, which reported that higher household meal frequency was significantly associated with a lower risk of underweight among lactating mothers [11,29,30,34]. Results from the KIIs support these findings, as they reported that increased meal frequency facilitates a good nutrition and normal weight status among lactating mothers. They noted that the more meals the lactating mother consumes, the greater the chances of consuming a variety of nutritious foods that meet the increased calorie and nutrition needs of the lactating mothers for a healthy life and sufficient quality breastmilk production. Hence, sensitization of the community on the need for lactating mothers to consume extra meals on top of the household meals could help improve the nutrition status of lactating mothers in the urban slums of Kampala City and other low socioeconomic status settings.

In the present study, lactating mothers who attained the MDD-W had a significantly lower prevalence of underweight. This could be because a mother who has achieved a MDD-W has consumed foods from at least five of the ten standard food groups designated as optimal dietary diversity the FAO [41]. A higher dietary diversity is correlated with better diet quality [64,65], the more diverse the food groups consumed by the mother are, the more likely she is to have consumed adequate and high-quality nutrients, including calories and essential micronutrients. These diverse food consumptions are necessary to meet her dietary needs for a good health and nutritional status, as well as for the production of sufficient and high-quality breastmilk [64,65]. Our findings are consistent with those of several previous studies conducted among lactating mothers in Ethiopia, which also reported that mothers who did not meet the MDD-W were at a significantly higher risk of being underweight, whereas those who attained the MDD-W had a significantly lower risk of being underweight [11,12,28–30,34–37]. Among the KII responses in our study, it was noted that meeting the MDD-W is a key factor in achieving a good nutrition and normal weight status among lactating mothers. The health workers stated that highly diverse diets include a variety of foods that provide the nutrients needed by lactating mothers to meet their daily dietary requirements and support breastmilk production. Therefore, community sensitization on the need for adequate dietary intake and dietary diversity among lactating mothers, with emphasis on consumption of locally available foods in season could help improve the nutrition status of lactating mothers in Kampala’s urban slums and similar areas.

### Study limitations

This study had some limitations. It was a cross-sectional study which made it difficult to establish a causal relationship between the outcome and exposure variables. Social desirability and recall biases might have affected some responses as they depended on the respondent’s memory. The absence of focus group discussions or in-depth interviews among lactating mothers also limited our ability to capture their perspectives and gain a nuanced understanding of their lived experiences in urban slum settings. However, several measures like quality control checks were incorporated in the questionnaire to ensure the accuracy of the data that was collected. The study also had some strengths, including the use of a mixed methods study design, that allowed for the triangulation of the quantitative findings with the qualitative findings to provide a comprehensive and better contextual understanding of the findings. The sample size was calculated using the single proportion formula by Kish [26] to address the prevalence estimation objective rather than a formula powered to detect a prespecified effect size for association analyses. Estimating the prevalence of underweight was one of the study objectives and informed the sample size determination. Consequently, the study may have had limited power to detect small associations. However, the sample size was increased to account for the design effect and anticipated non-response. Furthermore, the primary multivariable model was specified a priori, and findings remained consistent across sensitivity analyses, suggesting that the observed associations were robust.

## Conclusions

Underweight among lactating mothers remains a severe public health problem in Kampala’s urban slums. The independent correlates of underweight among lactating mothers were household food insecurity, household size of fewer than five members, daily household meal frequency of more than three meals per household per day, and minimum dietary diversity for women. Community sensitization on family planning, household food security, meal frequency, and dietary diversity through integrated community outreaches could improve the nutrition status of lactating mothers in the urban slums and other low socioeconomic status settings. Future research should examine the prevalence and correlates of underweight among adolescent lactating mothers to better understand its burden and determinants and inform targeted nutrition interventions in this vulnerable population.

## Supporting information

S1 Table Summary of the themes and representative quotes from key informant interviews on health workers’ perceptions of factors associated with a higher and lower prevalence of underweight among lactating mothers living in Kampala’s urban slums. (DOCX)

S1 Data Quantitative dataset. (XLS)
