# Supplementary material for: Prevalence and correlates of underweight among lactating mothers aged 20–49 years living in the Urban Slums of Kampala City, Uganda: A mixed-methods cross-sectional study
Source: PLOS Glob Public Health. 2026 Sep 29;6(9):e0007389. doi: 10.1371/journal.pgph.0007389 (PMC13623119; doi:10.1371/journal.pgph.0007389)
Supplement: Supplementary file 1 [file pgph.0007389.s001.docx]

**S1 Table. Summary of the themes and representative quotes from key informant interviews on health workers’ perceptions of factors associated with a higher and lower prevalence of underweight among lactating mothers living in Kampala’s urban slums.**

| **Themes** | **Description** | **Quote** | **Location** | **KII ID** |
| --- | --- | --- | --- | --- |
| Current state of underweight | Most key informants noted that the prevalence of underweight among lactating mothers living in the urban slums of Kampala Capital City was notably high and exceeded the national average. They emphasized that these mothers faced multiple, interrelated challenges that negatively affected their nutritional status, and made it difficult for them to maintain a healthy weight during the lactation period. | *“The prevalence of underweight among lactating mothers in the urban slums of Kampala Capital City is higher than the national average. These mothers face a complex range of challenges that hinder them from achieving adequate nutritional status.”* | Makindye Division Headquarters | KII-08 |
| Maternal related factors | One of the perceived facilitators mentioned by the key informants was eating frequent meals. They noted that eating frequent meals facilitated a good nutrition and normal weight status among lactating mothers. They noted that the more meals the lactating mother consumes, the greater the chances of consuming a variety of nutritious foods that meet the increased calorie and nutrition needs of the lactating mothers for a healthy life and sufficient quality breastmilk production. | *“I also believe that meal frequency is an important facilitator of normal nutritional status among lactating mothers. The more meals a lactating mother consumes, the greater the likelihood of meeting her increased calorie and nutrient needs for maintaining good health and producing sufficient, high-quality breastmilk.”* | Kawempe Division Headquarters | KII-04 |
|  | High educational attainment among lactating mothers was also identified by the key informants as a facilitator of good nutritional status. They noted that mothers who completed higher levels of education often gained knowledge about maternal nutrition during pregnancy and lactation, which helped them make better dietary choices. They also mentioned that such mothers were more likely to secure higher-paying jobs, which provided them with enough money to purchase the necessary foods to maintain a good nutritional status. | *“I also think that lactating mothers who have attained higher levels of education are less likely to be underweight. This could be because, at higher levels of education, they often gain knowledge about maternal nutrition during pregnancy and lactation, which helps them make better dietary choices. Additionally, mothers with higher education are more likely to have better-paying jobs compared to those with no formal education. This allows them to have enough money to buy all the foods they need to achieve a good nutrition status.”* (KII-05, Nakawa Division Headquarters) | Nakawa Division Headquarters | KII-05 |
|  | The key informants emphasized that achieving the minimum dietary diversity for women played a crucial role in promoting good nutritional status and maintaining a healthy weight among lactating mothers. They pointed out that although few lactating mothers were aware of the importance of meeting the dietary standard, some managed to achieve it unintentionally through varied diets. According to the informants, consuming a wide range of food groups helped ensure that lactating mothers received the essential nutrients required to meet their increased dietary needs and to produce sufficient and nutritious breastmilk. | *“Few lactating mothers are aware of the importance of meeting the minimum dietary diversity for women, though some achieve it unknowingly. Minimum dietary diversity is a key facilitator of good nutrition and a healthy weight status among lactating mothers, as diverse diets provide a variety of foods rich in the nutrients essential for meeting daily dietary needs and supporting breastmilk production.”* | Rubaga Division Headquarters | KII-01 |
|  | It was also mentioned by the key informants that being employed and working away from home were barriers to achieving a healthy weight and good nutritional status among lactating mothers, while staying at home was seen as a facilitator. They explained that working mothers, after dedicating time to their jobs and caring for their husbands and children, often had little or no time to care for themselves. In contrast, they noted that housewives were able to use the time they would have spent working to focus on their own well-being. | *“I also believe that maternal unemployment may, in some cases, be a blessing in disguise for housewives, as it can serve as a facilitator of good nutrition and healthy weight status among lactating mothers. This may be because housewives often have more time for self-care after attending to their husbands and other family members, unlike employed mothers who, after fulfilling work responsibilities and caring for their families, are often left with little or no time to focus on their own well-being.”* | Kampala Central Division Headquarters | KII-10 |
| Family and societal related factors | The key informants noted that access to quality health services, including antenatal and postnatal care, was a key facilitator of normal nutritional status among lactating mothers. They explained that mothers who attended all recommended antenatal and postnatal visits received valuable counselling, including on maternal nutrition during pregnancy and lactation. This knowledge helped them understand what foods to eat and how frequently, which improved the quality of their dietary intake and supported better nutritional outcomes. | *“It’s our duty to provide quality health services to our people. When the lactating mothers have access to quality health services, including antenatal care and postnatal care, and they attend all the recommended visits, they can receive valuable nutrition counselling during pregnancy and lactation. This helps them understand what foods to eat and how often, which improves the quality of their dietary intake and promotes healthy weight outcomes.”* | Rubaga Division Headquarters | KII-02 |
|  | The KII findings also indicated that having fewer household members was a facilitator of healthy nutritional status among lactating mothers. They noted that smaller household sizes reduce competition for food, whereas larger households increase competition, often leaving lactating mothers with less food since they typically serve themselves last after everyone else has eaten. | *“Another factor that facilitates a normal nutritional status among lactating mothers, particularly in households within urban slums, is having a small household size. Fewer household members reduce competition for food and other resources, allowing lactating mothers greater access to adequate meals. This enables them to meet their dietary needs, maintain a healthy nutritional status, and produce sufficient, nutritious breastmilk. Larger household sizes increase competition for food, limiting access for lactating mothers. Since they often prioritize caring for other household members, they tend to eat last and may receive less food, which predisposes them to underweight**”* | Nakawa Division Headquarters | KII-06 |
| Maternal related factors | The KIIs also noted that short birth intervals were a barrier to achieving normal nutritional status among lactating mothers. They explained that closely spaced pregnancies can lead to nutrient depletion, making it difficult for mothers to maintain a healthy weight while breastfeeding. They noted that each pregnancy draws from the mother’s nutrient reserves, and when conception occurs before the reserves are replenished, it increases their risk of becoming underweight. | *“I also think that short birth intervals can negatively affect a lactating mother’s nutritional status. Every pregnancy depletes a woman’s nutrient stores, and when she conceives again before fully recovering, it becomes difficult for her to maintain a healthy weight while breastfeeding. That’s why many mothers who have multiple deliveries in a short period are often underweight and unhealthy*.” | Makindye Division Headquarters | KII-07 |
|  | The KII findings also revealed that chronic illnesses such as Sickle Cell Disease, and HIV/AIDS were significant barriers to achieving a healthy weight and good nutritional status among lactating mothers. They explained that these conditions often reduce food intake due to symptoms like nausea, vomiting, or medication side effects that cause appetite loss. Additionally, they also noted that those illnesses can impair the absorption of nutrients through gastrointestinal symptoms like diarrhea and gastritis and increase the body’s nutrient demands because of heightened metabolic activity, all of which contribute to undernutrition. | *“Maternal chronic diseases such as Sickle Cell Disease, and HIV/AIDS can prevent lactating mothers from achieving a healthy weight and good nutritional status. These conditions may reduce food intake due to symptoms like nausea and vomiting or side effects of medications that cause loss of appetite. They can also impair nutrient absorption through symptoms such as diarrhea and gastritis, and increase the body’s nutrient requirements due to elevated metabolic demands."* | Kampala Central Division Headquarters | KII-09 |
|  | Recent abortion history was also identified by the key informants as one of the barriers to achieving normal nutritional status among lactating mothers. They mentioned that complications from abortion, such as anemia and infections like septicemia, can affect the intake and utilization of nutrients by the lactating mother, leading to underweight. | *“I personally think that having a recent abortion history is one of the barriers to achieving a normal nutritional status among lactating mothers. Abortions are usually complicated by anemia and infections like septicemia, which affect the intake and utilization of nutrients by the lactating mother, thereby predisposing her to underweight.”* | Kawempe Division Headquarters | KII-03 |
| Family and societal related factors | The KII findings also revealed that household food insecurity was a major barrier to achieving a healthy nutritional status among lactating mothers in the urban slums of Kampala Capital City. They noted that many households in these areas lack land to grow food and rely entirely on purchasing it. They mentioned that due to unemployment or low income, most families cannot afford adequate food, resulting in insufficient intake for lactating mothers and increasing their risk of becoming underweight. | *“Household food insecurity is a major barrier to achieving adequate nutrition among lactating mothers in the urban slums of Kampala Capital City. In these areas, residents typically lack space to grow their own food, making them entirely dependent on purchasing it. However, many have no employment or earn very low incomes, and the limited money they have is often insufficient to buy enough food for all household members. As a result, lactating mothers, like other members of the household, do not consume enough food, which puts them at risk of underweight.”* | Nakawa Division Headquarters) | KII-06 |
|  | The key informants also mentioned that having polygamous husbands was a barrier to normal nutritional status among lactating mothers. They noted that many women depend on their husbands for food, and in polygamous settings, the husband’s resources are divided among multiple households, which reduces the financial support each mother receives, increasing the risk of food insecurity and predisposing lactating mothers to underweight. | *“…., additionally, lactating mothers with polygamous husbands are more likely to be underweight. Many of these mothers rely on their husbands for food, but in polygamous households, the husband's resources are divided among multiple families. This reduces the financial support each woman receives, increasing the risk of food insecurity. On top of that, the competition and tensions with co-wives can cause psychological stress, which further contributes to poor nutritional status of the lactating mothers.”* | Kawempe Division Headquarters | KII-03 |
